# *Ascaris lumbricoides* Cystatin Prevents Development of Allergic Airway Inflammation in a Mouse Model

**DOI:** 10.3389/fimmu.2019.02280

**Published:** 2019-09-27

**Authors:** Sandra Coronado, Josefina Zakzuk, Ronald Regino, Velky Ahumada, Ines Benedetti, Alba Angelina, Oscar Palomares, Luis Caraballo

**Affiliations:** ^1^Institute for Immunological Research, Universidad de Cartagena, Cartagena, Colombia; ^2^Faculty of Medicine, Universidad de Cartagena, Cartagena, Colombia; ^3^Department of Biochemistry and Molecular Biology, Chemistry School, Complutense University of Madrid, Madrid, Spain

**Keywords:** *Ascaris*, recombinant cystatin, immunomodulation, allergy, IgE, dendritic cells, IL-10, Tregs

## Abstract

Severe helminth infections are negatively associated to allergic diseases like asthma; therefore, the immunomodulatory properties of parasite-derived components have been analyzed, raising the possibility of their use as anti-inflammatory molecules. We evaluated the immunomodulatory properties of *Ascaris lumbricoides* recombinant cysteine protease inhibitor (rAl-CPI) in a mouse model of allergic airway inflammation induced by the house dust mite (HDM) *Blomia tropicalis* and its effects on human monocyte-derived dendritic cells (HmoDCs). The *B. tropicalis* sensitized/challenged mice developed extensive cellular airway inflammatory response, which was significantly reduced upon treatment with rAl-CPI prior to *B. tropicalis* sensitization, affecting particularly the perivascular/peribronchial infiltrate cells, eosinophils/neutrophils, and goblet cells. A significant decrease of Th2 cytokines, total, and specific IgE antibodies was observed in rAl-CPI treated mice. The antibody response was biased to IgG, mainly IgG2a. Administration of rAl-CPI-alone and rAl-CPI before mite sensitization were associated with a significant increase of regulatory T cells (Tregs) in spleen and elevated IL-10 levels in BAL and splenocytes culture supernatants, which was partially affected by anti-IL10 receptor use. *In vitro*, rAl-CPI showed a modulatory effect on HmoDCs, lowering the expression of HLA-DR, CD83, and CD86, while inducing IL-10 and IL-6 production. This suggests an inhibition of HmoDC maturation and a possible link with the inhibition of the allergic response observed in the murine model.

## Introduction

In addition to their well-known detrimental effect on nutrition and children growth, helminth infections may modulate the host immune system and influence the pathophysiology of other immunological/inflammatory conditions (1, 2). *Ascaris lumbricoides* is a highly prevalent soil-transmitted helminthiasis among humans living in low-to-middle income countries. Although the immunity against this parasite involves a type 2 response characterized by high total and specific IgE and eosinophilia, *A. lumbricoides* produces molecules that modulate the host response toward a suppression state, creating an anti-inflammatory environment that promotes parasite survival (3, 4). In contrast to other helminths considered as strong immunosuppressors, ascariasis has been mainly recognized as an epidemiological risk factor for asthma presentation and severity, which could be biologically explained by the presence of IgE binding molecules cross-reacting with house dust mite (HDM) and other environmental allergens (5) and by its larval migration through the lung that permits a direct exposure to these allergenic molecules (6). However, this parasite is also able to down-regulate host immune responses. Chronically infected ascariasis patients with high parasite load have reduced cellular reactivity and lower type 1 cytokines TNF-α, IFN-γ, and IL-12 than non-infected endemic controls (7, 8). This immune hypo-responsiveness has been associated with increased spontaneous production of IL-10 and a modified Th2-like phenotype (9). Also, heavy infection has been associated with protection from asthma and atopy in rural settings (10). In this respect, the relationship between asthma and ascariasis is complex as immune suppression may depend on parasitic load (11). According to the current knowledge, in a context of low-intensity infection, the allergenic potential of *A. lumbricoides* overshadows the immune suppressor effects observed with heavy infections, probably leading to the positive associations between asthma and helminthiases reported by several groups (12).

The suppressive effect of *Ascaris* spp. somatic extracts and body fluid (ABF) on the humoral and cellular immune response has been well characterized using several animal models of inflammation, including allergic asthma (13–16). ABF, for example, suppresses the mucosal allergic inflammation by different mechanisms (not completely elucidated) that include the alteration of dendritic cell (DC) and macrophage function (17–20). However, information about the immunomodulatory capacity of purified excretory/secretory (E/S) products is scarce, with PAS-1 being the best-characterized protein. This protein modulates allergic airway inflammation via the induction of CD4^+^CD25^+^Foxp3^+^ T cells and IL-10/IFN-γ production (16, 21–23). With the genome sequencing of *Ascaris* species, a wide list of potential immunomodulators (based on homology with others identified in helminths) has been identified (24). Further characterization of these mediators is needed to understand the immunomodulatory potential of this parasite. Nonetheless, recently there is a growing interest for the mechanisms underlying helminth-induced immunomodulation by individual molecular mediators due to their therapeutic potential for inflammatory conditions (25).

In the case of *A. lumbricoides*, we focused on a cysteine protease inhibitor (or cystatin) whose enzymatic function may influence antigenic presentation and modulate innate and adaptive immune responses (26). Helminth cystatins play an important role in modulating host immunity and also protect from mucosal inflammation by different mechanisms, including inhibition of MHC-II expression and antigenic presentation by dendritic cells, increase of nitric oxide production and the induction of regulatory cytokines (IL-10 and TGF-β) as well as a regulatory profile in macrophages (27–30). Mei et al. confirmed that the 14.6 kDa product of *A. lumbricoides* with homology to other helminth cystatins is a functional active cysteine protease inhibitor with a typical tertiary structure expected for this protein family (31, 32). Recently, we reported that the recombinant cystatin of *A. lumbricoides* (rAl-CPI) induces high levels of IL-10 and TGFβ in a murine macrophage cell-line and in re-stimulated splenocytes, ameliorating inflammatory responses in a mouse model (33). Here, we aim to evaluate the ability of rAl-CPI to interfere with the development of allergic inflammation induced by a clinically relevant allergenic HDM (endemic in the tropics), in preventive settings, 4 h prior to sensitization with *Blomia tropicalis* extract. Since some *A. lumbricoides* components can induce an allergic response, we also explored the allergenicity of rAl-CPI with a similar sensitization/challenge protocol. In addition, we analyzed the immunomodulatory effect of rAl-CPI on monocyte-derived human DCs (HmoDCs).

## Methods

### Expression and Purification of rAl-CPI

The cDNA of *A. lumbricoides* cystatin was cloned into pQE30 vector (GenScript, NJ, USA) and expressed in *Escherichia coli* SG13009 strain. The recombinant product (rAl-CPI) was purified by affinity chromatography using a Ni-NTA column (Qiagen, Hilden, Germany) as described previously (33). A ToxinEraser™ column (GenScript, NJ; USA) was used for endotoxin removal; the final LPS concentration (0.0087 EU/mg) was quantified by a ToxinSensor Chromogenic LAL assay (GenScript, NJ, USA).

### Model of Allergic Airway Inflammation

**Figure 1:**
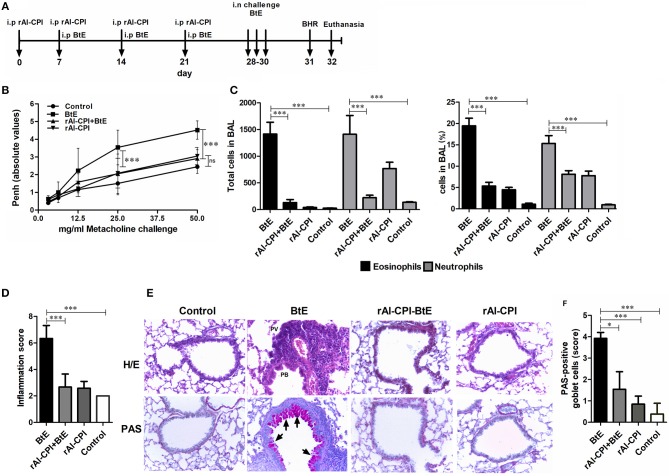
Influence of rAl-CPI on allergic airway hyper-reactivity and inflammatory infiltrate. **(A)** Scheme of the experimental protocol used in the animal model of allergic airways inflammation. **(B)** MCh induced airway reactivity was indirectly evaluated by whole body plethysmography. **(C)** Eosinophil and neutrophil numbers in BAL. **(D)** Total inflammation scores obtained from the histopathological analyses in lung tissue sections from each experimental group. **(E)** Representative photomicrographs (X100) from the different groups are shown. Areas with PV or PB inflammation are labeled in H/E stained slides (top row). **(F)** PAS-positive cells are observed in magenta (indicated by arrowheads). Mean values ± SEM are shown for 6–7 mice per group; representative data of three independent experiments. One way ANOVA + Dunnet, comparison with PBS and BtE controls, two-way ANOVA for methacholine concentration, Kruskall Wallis for inflammation score and Goblet cells. ^*^p ≤ 0.05; ^***^p ≤ 0.001.

Female (6–8-week-old) BALB/c mice were obtained from the National Institute of Health (Bogotá, Colombia) kept under pathogen-free environment (22°C, 50–60% humidity, 12 h light/dark cycle) and fed with standard pellet diet and drinking water *ad libitum*. Animals were sensitized three times (days 7, 14, and 21) via i.p. with 20 μg of *B. tropicalis* extract (LPS content: 2 EU/mg) emulsified in 2 mg of alum (Imject Alum, ThermoFisher Scientific, USA). On days 28, 29, and 30, mice were anesthetized with sevoflurane and challenged intranasally with 25 μg *B. tropicalis* extract. Naive controls were injected with alum in PBS and mock-challenged using PBS (25 μL per nostril) (34). The rAl-CPI-treated group additionally received i.p. 20 μg of this protein four times in weekly intervals, starting 1 week before sensitization (day 0) and 4 h before *B. tropicalis* extract injection (Figure 1A). To study the response to rAl-CPI alone, mice were sensitized and challenged with 20 μg of the protein in a similar protocol to that described for *B. tropicalis* extract. In addition, two additional groups were injected i.p with 100 μg of a neutralizing anti-mouse CD210 monoclonal antibody (anti IL-10R) or 100 μg of IgG isotype control (eBioscience, San Diego, CA, USA) (35, 36) prior to rAl-CPI injection and/or allergen sensitization. Animal and human experiments were approved by the Ethical Committee of University of Cartagena (Minute 36, 13-10-2011) and were performed in accordance with institutional protocols and international regulations. Additionally, for human experiments, peripheral blood mononuclear cells (PBMC) were obtained from buffy coats of healthy donors from the Centro de Transfusión de la Comunidad de Madrid.

### Measurement of Airway Responsiveness

Airway responsiveness was determined on day 31 by whole body plethysmography (Buxco Electronics, Troy, N.Y., USA) in unrestrained animals by provocation with increasing doses of methacholine (MCh) (Sigma–Aldrich, Germany) as described elsewhere (34). Changes in Penh (enhanced pause) values were used as an indirect measure of airway hyper-reactivity (37, 38).

### Bronchoalveolar Lavage (BAL)

BAL was harvested by flushing the lung airways via the trachea (2X) with 0.8 mL of ice-cold PBS containing a complete protease inhibitor cocktail (Roche, Germany). After centrifugation for 10 min at 340 g and 4°C, supernatants were collected and stored at −80°C for subsequent cytokine measurements. Immediately after obtaining the BAL, samples were processed and CD45+ cells, alveolar macrophages (Siglec-F^+^CD11c^+^), eosinophils (Siglec-F^+^CD11c^−^), and neutrophils (CD11b^+^Ly6G^+^) were identified by cell surface marker expression (39, 40). Fluorochrome-conjugated monoclonal antibodies and gating strategy are shown in Supplementary Table 1 and Supplementary Figure 1.

### Cytokine Splenocytes Profile

A single cell suspension of spleen was obtained after tissue homogenization and cell viability was measured by Trypan Blue (0.4%) exclusion using an automated cell-counter (TC20 BioRad, United States); in all cases, the viability was >90%. Splenocytes were cultivated in RPMI-1640 medium supplemented with 1 mM sodium pyruvate, 2 mM, L-glutamine, 100 U/mM penicillin-streptomycin, and 10% heat-inactivated fetal bovine serum (FBS, Qualified One Shot, Gibco, USA). Cells (1 × 10^6^) were stimulated with 20 μg/mL *B. tropicalis* extract, 20 μg/mL rAl-CPI or medium alone and incubated at 37°C and 5% CO_2_ for 72 h. Cell culture supernatants were stored at −80°C until use. Cytokine levels (IL-4, IL-5, IL-13, IL-10, IFNγ, and TGF-β) were determined by ELISA, using commercial kits (eBiosciences, USA).

Tregs were identified in the cell suspension as CD3^+^CD4^+^CD25^+^Foxp3^+^ using fluorochrome conjugated antibodies (Supplementary Table 1 and Supplementary Figure 2), following the instructions of the anti-mouse Foxp3 staining kit (BD, San Diego, CA, USA). Cells were identified in a FACSAria III (BD Biosciences, USA) and analyzed using Kaluza Analisis version 2.1 (Beckman Coulter, USA).

### Antibody Analyses

Total IgE, IgG, IgG1, and IgG2a levels were determined using commercial kits, following manufacturer's instructions (Ready-Set-Go kits, eBioscience, San Diego, CA, USA). For specific immunoglobulin determinations, Maxisorp™ microtiter plates were coated with *B. tropicalis* extract at 5 μg/mL by overnight incubation at 4°C and washed 4 times with Tween 20 0.1% PBS. Wells were then blocked with PBS 1% BSA 0.05% Tween 20 for 3 h at room temperature. Plates were washed (5X) and incubated overnight with diluted mouse plasma samples at 4°C. After 5 washes, wells were incubated for 1 h at RT with biotin labeled anti-mouse IgE, IgG1, or IgG2a. After 5 washes, ExtrAvidin alkaline-phosphatase (Sigma-Aldrich, Saint Louis, Missouri, USA) was added and incubated an additional hour. Paranytrophenil diphosphate (1 mg/mL) was used as substrate solution. Optical densities were read at 405 nm in a spectrophotometer. To increase the sensitivity of ELISA/IgE, IgG was depleted from plasma by incubation with protein G sepharose (41). Dilutions of samples and detection antibodies are described in the Supplementary Table 2.

### Histological Analyses

Lungs were immersed in 10% neutral-buffered formalin. For processing, lung tissues were first embedded in paraffin, cut into 4 μm thick sections for hematoxilin-eosin (H&E) and Periodic Acid-Schiff (PAS) staining. Slides were visualized by light microscopy to evaluate lung inflammation and mucus production. To determine the severity of inflammatory cell infiltration, peribronchial (PB), and perivascular (PV) inflammatory cell counts were performed blind as described elsewhere (34). Images were captured at ×400 and ×100 final magnification on an Eclipse 400 microscope connected to a DS-Fi1m camera (Digital camera Nikon, Japan). Images were processed with NIS-Elements-3.01 software (Nikon Elements Software).

### Effects of rAl-CPI Over Human Monocyte-Derived Dendritic Cells (HmoDCs)

PBMCs from healthy donors were isolated by using Ficoll-Paque Plus (GE Healthcare, UK) density gradient centrifugation from heparinized blood. Monocytes were isolated using anti-human CD14 microbeads by positive magnetic selection in autoMACS (Miltenyi Biotec, Germany) according to the manufacturer's recommendations. Purified monocytes were cultured in RPMI containing 10% FBS and recombinant human GM-CSF and IL-4 (100 ng/mL) as previously described (42). After 6 days of culture, immature HmoDCs were harvested (2 × 10^5^ cells/well) and stimulated with LPS (100 ng/mL, Sigma-Aldrich, USA), rAl-CPI-only (100 ng/mL), or incubated with rAl-CPI or PBS as a control 30 min prior to the addition of LPS (43). The expression of surface markers was assessed using anti-human HLA-DR–FITC, CD86-PE, and CD83-APC (Miltenyi Biotec, Bergisch Gladbach, Germany). Cell viability was routinely checked by using trypan blue cell exclusion in the light microscope and propidium iodide staining in flow cytometric analysis (Supplementary Figure 3). CD4^+^CD45RA^+^CD45RO^−^ naïve T cells were purified from PBMCs by negative selection using CD4 T cell isolation kit (Miltenyi Biotec, Bergisch Gladbach, Germany). T cell subset purity was routinely above 92%. HmoDCs were used to stimulate allogeneic CD4^+^ T cells by co-culture (1:5) and cytokines produced after 6 days of coculture were measured by ELISA.

### Statistics Analyses

Data were expressed as mean values with the standard error of the mean (SEM), unless otherwise indicated and analyzed using the GraphPad Prism 4.0 statistical software. Statistical analysis included Student's *t*-test and one-way ANOVA with *post-hoc* test analysis or the Kruskal–Wallis test when appropriate. *P* ≤ 0.05 were considered statistically significant.

## Results

### rAl-CPI Reduces Airway Hyper-Reactivity (AHR) and Inflammation in the Lungs

In order to evaluate the effect of rAl-CPI on the development of airway allergic response, rAl-CPI was administered before the HDM sensitization/challenge protocol (Figure 1A). *B. tropicalis* extract administration induced significantly higher Penh values, in response to different doses of methacholine suggesting airway hyper-reactivity and different manifestations of lung inflammation. BAL cellular content was rich in neutrophils (~15%) and eosinophils (~20%). Treatment with rAl-CPI significantly reduced Penh values (Figure 1B) and the lung inflammatory response induced by *B. tropicalis* extract, including eosinophils and neutrophils in BAL (Figure 1C). Lung histological analysis confirmed reduced lower airway cell infiltration and goblet cell hyperplasia (Figures 1D–F). Administration of rAl-CPI alone failed to induce significant changes in Penh values, but elicited a predominant neutrophilic inflammation (~7%) that was higher than the control group, but significantly lower than that obtained with the *B. tropicalis* extract (Figure 1C).

### rAl-CPI Inhibits the Production of Total IgE and Specific IgE to *B. tropicalis* Extract, Promoting IgG Response

**Figure 2:**
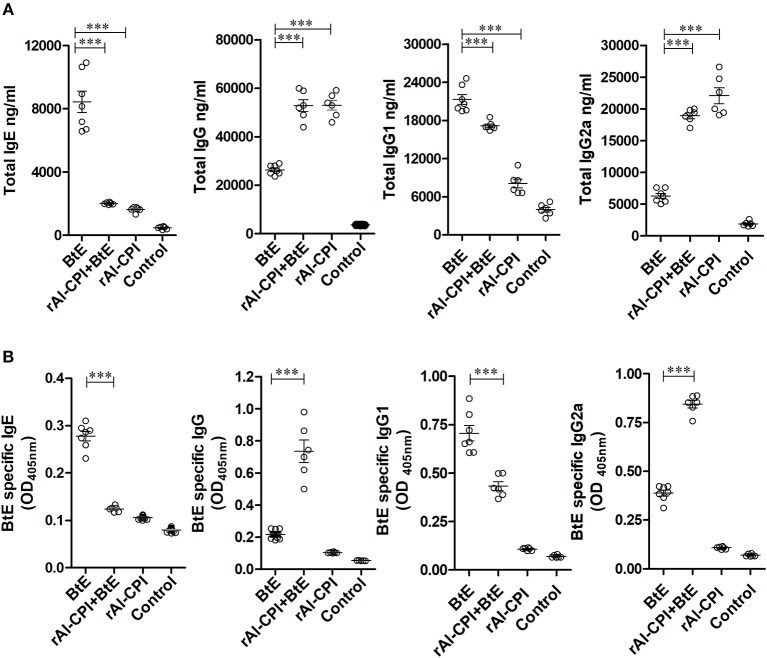
rAl-CPI interferes with IgE production and potentiates IgG2a response. **(A)** Serum Total IgE, IgG, IgG1, and IgG2a antibody levels. **(B)** Serum BtE allergen-specific IgE, IgG IgG1, and IgG2a antibody levels. The mean values ± SEM are shown for 6–7 mice per group; representative data from three independent experiments. One-way ANOVA with Dunnet *post-hoc* analysis). ^***^p ≤ 0.001.

*Blomia tropicalis* extract administration increased total IgE, IgG, IgG1, and IgG2 antibodies compared to the control group. In contrast, treatment with rAl-CPI/*B. tropicalis* extract significantly reduced total IgE and IgG1 responses, but increased the total IgG2a response (Figure 2A). In addition, treatment with rAl-CPI/*B. tropicalis* extract inhibited specific IgE and IgG1 to *B. tropicalis* while increasing the specific IgG2a antibody response (Figure 2B).

Administration of rAl-CPI alone did not raise total IgE production in comparison with the negative control; however, it increased total IgG production and the IgG2a response was even greater than that induced by *B. tropicalis* extract (Figure 2A). The specific antibody response to rAl-CPI was characterized by elevated levels of IgG2a and absence of specific IgE (Supplementary Figure 4).

### rAl-CPI Modifies Local Cytokine Production

**Figure 3:**
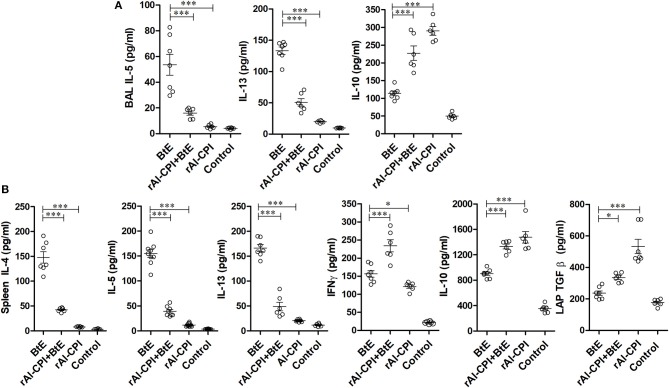
rAl-CPI modifies local and systemic cytokine responses. **(A)** Th2 cytokines detected in BAL: IL-5, IL-13, and IL-10. **(B)** Cytokines in spleen cell cultures restimulated *in vitro* with BtE (for group rAl-CPI culture was stimulated with rAl-CPI), IL-4, IL5, IL-13, IFNγ, IL-10, and LAP-TGFβ. The mean values ± SEM are shown for 6–7 mice per group; representative data from three independent experiments. One-way ANOVA with Dunnet *post-hoc* analysis. ^*^p ≤ 0.05; ^***^p ≤ 0.001.

To evaluate the rAl-CPI-mediated inhibition of the local immune responses against allergens, BAL cytokine levels were measured. IL-5 and IL-13 were significantly greater in *B. tropicalis* extract sensitized mice than in those receiving PBS. The rAl-CPI/*B. tropicalis* extract treated group showed significantly lower IL-5 and IL-13, but higher IL-10 production compared with the *B. tropicalis* extract sensitized group (Figure 3A). Treatment with rAl-CPI alone did not induce IL-5 and IL-13 production, however IL-10 levels were significantly higher than those detected in mice exposed to *B. tropicalis* extract. Measurement of cytokine levels in splenocyte culture supernatants indicated that systemic effects of rAl-CPI resembled those observed locally. Mice receiving rAl-CPI/*B. tropicalis* extract showed a marked reduction in IL-5, IL-13, and IL-4 production by splenocytes re-stimulated with *B. tropicalis* extract. Significant increase in IL-10, LAP-TGFβ, and IFNγ production were observed in the rAl-CPI-*B. tropicalis* extract group compared to the *B. tropicalis* extract group (Figure 3B).

### rAl-CPI Increases Tregs

**Figure 4:**
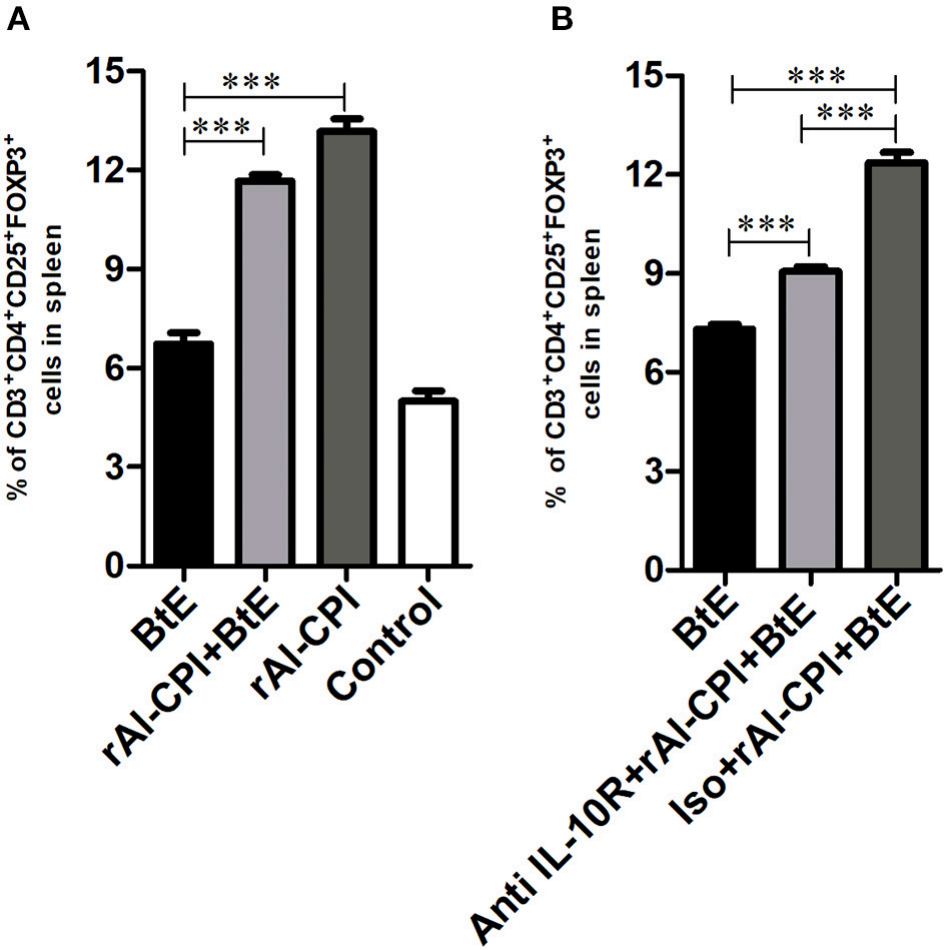
rAl-CPI stimulates Treg differentiation. **(A)** Percentage of Treg cells in spleen. **(B)** Percentage of Treg cells when the anti-IL-10R monoclonal or isotype control was used. The mean values ± SEM are shown for 6–7 mice per group; representative data from three independent experiments. One-way ANOVA with Dunnet *post-hoc* analysis. ^***^p ≤ 0.001.

Mice sensitized with *B. tropicalis* extract displayed higher percentages of Tregs (CD4^+^CD25^+^Foxp3^+^) in spleen compared to the PBS control group (7.25 vs. 5.56%, *p* < 0.001, Figure 4A). However, the frequency of Tregs was significantly increased in rAl-CPI/*B. tropicalis* extract treated mice than in those treated with *B. tropicalis* extract alone (11.6 vs. 7.25%, *p* = 0.05, Figure 4A). Remarkably, rAl-CPI administration alone induced the highest percentage (13.1%) of Tregs among all groups analyzed.

### Inhibition of IL-10 Signaling Pathway Reduces the Effect of rAl-CPI on Humoral Response Against *Blomia tropicalis*

**Figure 5:**
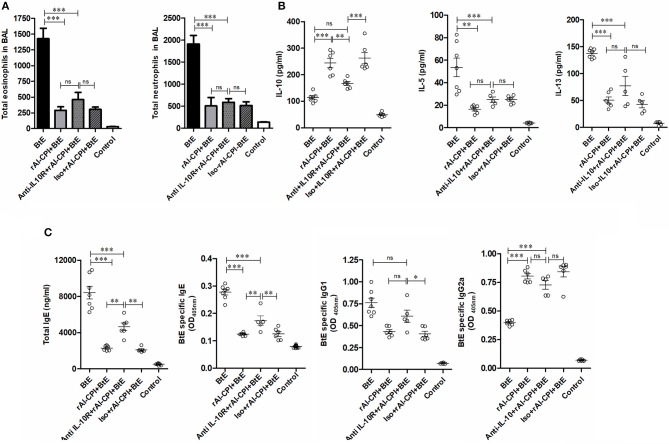
Inhibition of IL-10 signaling pathway reduces the effect of rAl-CPI on humoral response against *Blomia tropicalis*. **(A)** Total number of eosinophils and neutrophils in BAL. **(B)** Cytokine levels BAL, IL-10, IL-5, and IL-10. **(C)** Serum Total IgE, IgG1, and BtE allergen-specific IgE, IgG1, and IgG2a antibody levels. The mean values ± SEM are shown for 5–6 mice per group when the anti-IL-10R monoclonal was used. One-way ANOVA + Bonferroni for *post-hoc* comparisons). ^*^p ≤ 0.05; ^**^p ≤ 0.01, ^***^p ≤ 0.001.

The effect of IL-10 signaling pathway on the anti-inflammatory effects of rAl-CPI were evaluated by means of the systemic administration of anti-IL10R blocking antibody. As observed in Figure 4B, anti-IL10R mAb reduced the effect of rAl-CPI/*B. tropicalis* extract on Treg induction as compared with the isotype control. Blockade of IL-10R did not alter the inhibition of rAl-CPI treatment on IL-5 and IL-13 production or its preventive effects on pulmonary allergic inflammation in terms of the total number of eosinophils and neutrophils detected in BAL. However, it was observed that IL-10 levels in BAL (Figures 5A,B) were lower in sensitized mice that received rAl-CPI with anti-IL-10R compared to those treated with the isotype control instead. Total IgE and *B. tropicalis*-specific IgE/IgG1 antibodies were significantly higher in *B. tropicalis* exposed mice receiving rAl-CPI/anti-IL10R than in those injected with isotype control; however, this increase was not enough to reach antibody levels observed in mice sensitized and challenged with *B. tropicalis* (Figure 5C). Cytokine levels on splenocyte cultures did not significantly change with IL-10R administration. Although IL-10 levels were lower in mice receiving the blocking Ab, this reduction was not significant (Supplementary Figure 5).

### rAl-CPI Interferes the Maturation of Dendritic Cells

**Figure 6:**
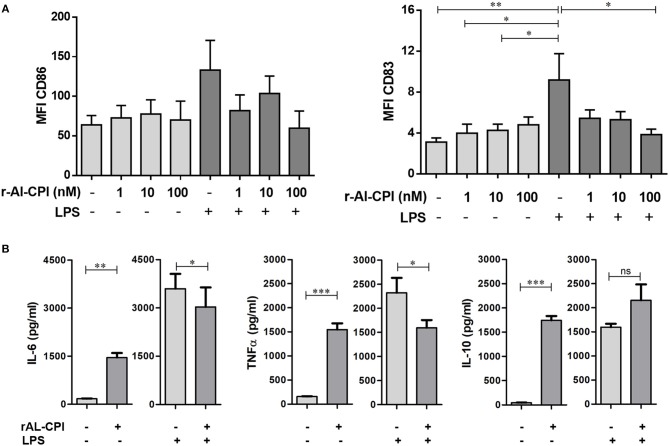
Effects of rAl-CPI on surface molecules expression and cytokine production by HmoDCs. HmoDCs isolated from five different donors were stimulated with LPS, rAl-CPI alone o rAl-CPI+LPS and analyzed for **(A)** cell surface marker expression and **(B)** cytokine production. Data presented are means of duplicate. One-way ANOVA with Dunnet *post-hoc* analysis for MFI data and *T*-test for cytokine comparisons. ^*^p ≤ 0.05; ^**^p ≤ 0.01, ^***^p ≤ 0.001.

In order to assess whether rAl-CPI exerts its immunomodulatory effect not only in the murine model, but also in the human system, the effect of rAl-CPI was tested in HmoDCs. As shown in Figure 6A, rAl-CPI treatment of immature HmoDCs did not significantly induce co-stimulatory molecules, CD86 and CD83 in HLA-DR+ HmoDCs compared to the control. After LPS stimulation, a significant increase in the expression of CD86 and CD83 was observed compared with controls, suggesting HmoDC maturation. Interestingly, rAl-CPI tended to reduce the expression of the co-stimulatory molecules CD86 and CD83 in LPS-activated HmoDCs. In respect to cytokine responses, although rAl-CPI treatment induced the production of IL-6/TNFα in immature HmoDC compared to controls, cytokine levels remained generally lower than in LPS-stimulated cells, which marginally increased IL-10 secretion in the presence of rAl-CPI (Figure 6B). IL-12 production was not detectable in any of the analyzed conditions, including LPS stimulation (data not shown). Significantly higher levels of IL 10 and IFNγ were detected in the co-cultures where the dendritic cells were previously stimulated with LPS, rAl-CPI, and rAL-CPI+LPS (Supplementary Figure 6).

## Discussion

Allergic diseases are worldwide public health problems that require new treatment approaches. For this reason, their complex relationship with helminth infections has become a matter of study during last years. Helminthiasis have a dual effect on the host immune response: depending on their intensity and host genetics, they may induce type 2 responses, but also an immunosuppressive state that may help prevent atopic disorders (12). Therefore, there is great interest in identifying excretory/secretory products of helminths with immunomodulatory potential. According to the *A. suum* genome and proteome, it is predicted that the genus *Ascaris* contains more than 15 products with these properties (24, 44). In this study, we explored the preventive effects of *A. lumbricoides* cystatin on airway inflammation induced by the HDM *B. tropicalis*, one of the most clinically relevant HDM in the tropics. For the first time, here we show that, similar to other helminth cystatins (36, 45, 46), the *A. lumbricoides* cystatin reduces HDM induced-allergic airway inflammation, airway hyper-reactivity (as detected by Penh values), and hallmarks of allergic inflammation after *B. tropicalis* sensitization, including eosinophil airways infiltration, goblet cell hyperplasia and elevated Th2 cytokine/IgE production.

In this model, rAl-CPI was administered 4 h before sensitization, successfully reducing allergic inflammation. This agrees with the observation that rAl-CPI induced an immunoregulatory response that included IL-10 and TGF-β production as well as a strong IgG2 response that may dampen the allergenic effects of the HDM *B. tropicalis*. Due to its cysteine protease inhibitory activity (27, 28), rAl-CPI may interfere with antigenic presentation and cytokine production by innate immune cells (DCs, monocytes, and macrophages) at an early stage. Administration of rAl-CPI *in vivo*, alone or with *B. tropicalis*, caused a significant elevation of IFNγ together with the immunoregulatory cytokines, a result also reported for PAS-1 (21). Similarly, we had observed that *in vitro* stimulation of mouse peritoneal macrophages and splenocytes with rAl-CPI induced a parallel increase of IL-10, TGFβ, and IFNγ (33). This scenario seems to be convenient to counteract the development of allergic responses. The role of IFNγ in the suppression of allergic asthma has been reported by others. In the work of Kim et al. it was observed that administration of crude extract of *Caenorhabditis elegans* in OVA sensitized mice reduced IL-4, IL-5, and IL-13 levels in BAL and increased IFNγ. Interestingly, the suppressive effect of this helminth preparation was abolished in IFNγ knockout mice (47). This microenvironment of IFNγ/IL-10 could directly diminish the IL-4 and IL-13 production, leading to downregulation of the IgE synthesis (48, 49). Also, it has been observed that the administration of bacterial antigens, that strongly stimulate Th1 response, inhibits the Th2 response to fungal allergens in murine models of allergic asthma (50); similarly, when CpG was administered before OVA sensitization, mice allergic airway inflammation was inhibited (51).

As observed here with HmoDCs and previously with murine macrophages (33), rAl-CPI, as other cystatins (28), induces strong IL-10, and TGFβ production. Particularly for IL-10, its well-known anti-allergic effects include functional alteration of antigenic presentation, inhibition of T cell responses, stimulation of IgG4 by human B cells or IgG2a (in mouse) and promotion of Treg cell development (52–54). IL-10 was proposed to be a major mechanism of protection from airway allergic inflammation in the case of cystatin from *Acanthocheilonema vitae* (36), hence, we sought to evaluate if this was the case also for rAl-CPI in this model of allergic inflammation. We found that IL-10R blockade reduced Treg cell numbers and the local IL-10 production in the lung, while significantly increasing type 2 humoral responses. However, IL-10R blockade did not fully counteract the effects of rAl-CPI on local airway inflammation, suggesting that other mechanisms may be involved in rAl-CPI–mediated modulation of allergic responses induced by *B. tropicalis*. This is contrast with the results of Schnoeller et al. showing that IL-10R blockade did restore the reduced number of infiltrating cells caused by the *A. vitae* cystatin in a murine model of OVA-induced allergic airway responsiveness (36). Although the observed results could be due to an incomplete blockade of IL-10R, it is worth mentioning that, according to other ascariasis or helminthiasis studies, IL-10 is not essential for host immunosuppression. For example, in an ovoalbumin (OVA) airway sensitization model, administration of pseudocelomic fluid of *A. suum* prevented allergic inflammation even in IL-10^−/−^ mice (17). Similar results were obtained with *Heligmosomoides polygirus* extract in a model where mesenteric lymph node cells from IL-10-deficient animals transferred suppression of allergic response to sensitized hosts (35).

The role of other immunoregulatory mechanisms that are stimulated by rAl-CPI (TGF-β and Tregs) deserves further investigation. It has been demonstrated for several helminth species that Tregs are necessary for suppressing allergic airway inflammation, in mice (55–60). Treatment of Foxp3^+^ Tregs with AIP-2 (a purified product of *Ancylostoma caninum*), completely abrogated the protection against eosinophilia and lymphocytes infiltration of the airways, in mice (57). In contrast, in the model airway allergy induced with *A. vitae* cystatin, Treg depletion with an anti-CD25 antibody partially reduced the efficiency of the filarial product to inhibit inflammation. In the case of rAl-CPI, it is necessary to evaluate anti-inflammatory effects in absence of Tregs (36). Compared with isotype control administration, IL-10R blockade reduced Treg cell frequency in spleen by about 30%, which is not enough to study rAl-CPI immunosuppressive function in the absence of Tregs. Further, HDM administration causes extensive epithelial damage, and oxidative stress in the airways (61, 62). Under these conditions, IL-10 signals might not be sufficient to resolve the inflammation and other cytokines may participate in the resolution of inflammation, including perhaps TGFβ (that we found increased upon rAl-CPI treatment).

As a cysteine protease inhibitor, rAl-CPI may theoretically block the enzymatic activity of potent cysteine proteases present in HDM extracts [as reviewed for Der p 1 (63)] and, in turn, reduce HDM pro-allergenic effects. However, we consider that in this sensitization model with *B. tropicalis*, this may be less important because cysteine protease levels are low in HDM species (64, 65).

Another important finding was that pre-administration of rAl-CPI restrained the total and HDM-specific IgE/IgG1 responses induced upon *B. tropicalis* sensitization, while increasing HDM-specific IgG/IgG2a responses. A previous work by Suzuki et al. reported contrasting results on HDM-specific IgE/IgG1 responses using *A. lumbricoides* complete extract before HDM sensitization (66). These differences might be justified by the presence of HDM-cross-reactive allergens in whole-body extracts (5). In other models of airway inflammation using OVA as sensitizer, administration of complete nematode extracts, such as *C. elegans* and *Marshallagi marshalli*, diminished the IgE and IgG1 responses and induced elevated IgG2a levels (47, 67). We found similar results for the purified rAl-CPI, also in agreement with previous data on other *Ascaris* spp. antigens, such as As37, As14, and As24 (68). We speculate that rAl-CPI induces itself high titers of IgG2a after sensitization and promotes mostly IgE to IgG isotype responses directed against the allergens of the extract. In this respect, the administration of purified immunosuppressive products such as rAl-CPI, could be a better therapeutic approach to prevent allergic inflammation. As mentioned, the inhibitory effect of rAl-CPI on *B. tropicalis*-specific IgE responses may be related to the decrease of IL-4 and IL-13, strictly required for switching from IgG to IgE (69). However, our results support the additional involvement of IL-10, a potent suppressor of total and allergen-specific IgE responses, as seen in immunotherapeutic protocols used in murine models of asthma (70).

As a translational approach, using human monocyte derived DCs, we explored if rAl-CPI modulates DC function and maturation similarly to other cystatins, such as the *H. polygyrus* cystatin (Hp-CPI) (71) (Hp-CPI). *In vitro*, the recombinant rHp-CPI modifies the phenotype and function of DC, leading to MHC-II and CD40/CD86 reduction, required for proper T-cell priming (72). In our study, after rAl-CPI treatment HmoDCs maintained an immature phenotype, even when the cells were stimulated with LPS, which is similar to that found using *F. hepatica* cystatin (43). It is possible that rAl-CPI inactivates the lysosomal cathepsins necessary for the HLA-DR processing (27, 73), as demonstrated for cystatin Bm-CPI-2 from *B. malayi* (30) and HcCyst-3 from *H. contortus* (74). It was previously reported that rAl-CPI inhibits different cathepsins and it was proposed that this underlies its immunomodulatory properties (32). A limitation of this study is that the impact of DC modulation by Al-CPI on allergen specific T cell response was not sufficiently evaluated. To address this, future studies are needed to investigate co-cultures of rAl-CPI-pulsed DCs with T cells and evaluate cell proliferation and cytokine production after allergen exposure. There is generally little information about the effects of cystatins on human adaptive immune responses. On the other hand, although Tregs increased upon rAl-CPI treatment, a thorough exploration of Tregs in local anatomical niches, such as the mesenteric lymph nodes and other affected tissues, is needed in future studies. Daniłowicz et al. reported that stimulation of PBMCs derived from grass pollen (GP) allergy patients with filarial cystatin, prior to GP extract exposure, reduced Th2 cytokine production. Also, filarial cystatin reduced CD4+ T cell proliferation to an unspecific stimuli (45).

In our study, we asked whether rAl-CPI immunomodulation prevents allergic airway response to HDMs endemic in the tropics, recapitulating natural exposure by anticipating helminth antigen exposure relative to the HDM challenge. In fact, we assumed that helminth exposure occurs early in life in high-risk populations, preceding the development of allergic asthma.

There is evidence that heavy infestation by *A. lumbricoides* reduces the risk of asthma presentation (10) and consistently in our model, we observe that early administration of a purified product of *A. lumbricoides* actually reduces *B. tropicalis* induced allergic inflammation. However, it is necessary to evaluate the therapeutic performance of rAI-CPI in other administration protocols, i.e., after allergen sensitization or challenge. For the *A. vitae* cystatin, suppression of the allergic response was reported when administered prior to intranasal challenges with OVA (pre-challenge model) (36). Also, other routes of administration must be explored since intraperitoneal injections are less likely to be convenient for human applications. In line with this, there is evidence that the filarial cystatin may prevent Th2 mediated airway disease by intranasal administration (75).

Our results reveal that, unlike other cystatins, rAl-CPI is poorly allergenic. The nasal challenge with rAl-CPI did not induce an eosinophilic inflammatory response in airways, neither increased Penh values as observed with the *B tropicalis* extract. Also, rAl-CPI specific IgE levels were undetectable in sensitized/challenged mice perhaps justifying the failure of rAl-CPI to increase airway reactivity (supported by the Penh values) and induce passive cutaneous anaphylaxis, in our previous work (76). Concerns about the allergenic potential of cystatins are raised by the recognition of the *Anisakis simplex* cystatin as an allergen (Ani s 4) (77) as well as others reported in kiwi and cat (78, 79). Although *A. vitae* cystatin is considered a strong immunosuppressive helminth product, its native form can stimulate β-hexosaminidase release in rat basophil leukemia cells sensitized with sera from immunized gerbils (80).

Another limitation of our study is the lack of a negative control to evaluate the impact of enzymatic function of rAl-CPI on the observed results. Heat-denaturation to alter the enzymatic function was not suitable in this case since rAl-CPI, as other cystatins, is highly thermostable (unpublished observation). Moreover, cystatin inhibitors are neither currently available. On the other hand, although Tregs in spleen are raised by rAl-CPI, exploration of regulatory cell populations must be complemented with local analysis in affected tissue and mesenteric lymph nodes.

In conclusion, our study demonstrates that the cystatin from *A. lumbricoides* reduces the development of HDM induced allergic airway inflammation and systemic (cellular and humoral) Th2-responses in mice, while impairing human DC maturation.

## Data Availability Statement

All datasets generated for this study are included in the manuscript/Supplementary Files.

## Ethics Statement

Animal and human experiments were approved by the Ethical Committee of the University of Cartagena (Minute 36, 13-10-2011) and were performed in accordance with institutional protocols and international regulations.

## Author Contributions

LC, JZ, and SC designed the study, supervised the experiments, analyzed results, and wrote the manuscript. OP and AA supervised dendritic cells experiments and contributed to manuscript writing. IB performed the histological analyses. RR contributed to recombinant cystatin production and animal model experiments. VA participated in animal model experiments. All authors revised the manuscript.

### Conflict of Interest

The authors declare that the research was conducted in the absence of any commercial or financial relationships that could be construed as a potential conflict of interest.

## Abbreviations

ABF: *A. lumbricoides* body fluid
AIP-2: Ancylostoma caninum anti-inflammatory protein 1
Ani s 4: Anisakis simplex cystatin allergen
anti-IL10R: anti-IL10 receptor monoclonal antibody
BAL: Broncho-alveolar lavage
BALB/c: Bagg albino mouse
CpG: Bacterial oligodeoxynucleotides
DC: dendritic cell
Der p 1: Dermatophagoides pteronyssinus antigen P1 cysteine protease
E/S: excretory/secretory products
EU/mg: Endotoxin units per milligram
FBS: Fetal bovine serum
FOXP3: forkhead box P3 transcription factor
GM-CSF: Granulocyte Macrophage Colony-Stimulating Factor
GP: Grass pollen
H&E: Hematoxilin-Eosin stain
HDM: House dust mite
HLA-DR: Human Leukocyte Antigen—DR isotype
HmoDCs: Human monocyte-derived dendritic cells
i.n.: intranasal administration
i.p.: intra-peritoneal administration
IFN-γ: Gamma interferon
IgE: Immunoglobulin E
IgG: Immunoglobulin G
IgG2a: Immunoglobulin G2a isotype
IL-10: Interleukin 10
IL-12: Interleukin 12
LAL: Limulus amebocyte lysate
LPS: Bacterial lipopolysaccharides/Endotoxin
OVA: Ovoalbumin
PAS: Periodic Acid-Schiff staining
PAS-1: A. suum-derived protein 1
PB: Peribronchial
PBMC: Peripheral blood mononuclear cells
PV: Perivascular
rAL-CPI: *Ascaris lumbricoides* recombinant cysteine protease inhibitor
TGFβ: Transforming growth factor beta
TNF-α: Tumor necrosis factor
Tregs: Regulatory T cells.
