# Supplementary material for: Ascaris lumbricoides Cystatin Prevents Development of Allergic Airway Inflammation in a Mouse Model
Source: Front Immunol. 2019 Sep 27;10:2280. doi: 10.3389/fimmu.2019.02280 (PMC6777510; doi:10.3389/fimmu.2019.02280)
Supplement: Supplementary file 1 [file Table_1.DOCX]

Supplementary Material

***Ascaris lumbricoides* cystatin prevents development of allergic airway inflammation in a mouse model**

Coronado S^1^, Zakzuk J^1^, Regino R^1^, Ahumada V^1^, Benedetti I^2^, Angelina A^3^, Palomares O^3^, Caraballo L^1^

*** Correspondence:** Corresponding Author: lcaraballog@unicartagena.edu.co

# Supplementary Tables

# Table 1 Antibodies used for flow cytometry

| **Flow cytometry Reactivity** | **Conjugate** | **Clone** | **Reference** | **Company** |
| --- | --- | --- | --- | --- |
| Anti mouse CD3 | PE-cyanine7 | 145-2C11 | 25-0031 | eBioscience |
| Anti mouse CD4 | FITC | GK1.5 | 11-0041 | eBioscience |
| Anti mouse CD25 | APC | PC61.5 | 17-0251-81 | eBioscience |
| Anti mouse FoxP3 | PE | FJK-16s | 12-5773-80 | eBioscience |
| Anti mouse CD11c | FITC | N418 | 11-0114 | eBioscience |
| Anti mouse CD11c | APC | HL3 | 550261 | BD Pharmigen |
| Anti mouse CD45 | eFluor450 | 30-F11 | 48-0451 | eBioscience |
| Anti mouse CD45 | FITC | 30-F11 | 553080 | BD Pharmigen |
| Anti mouse CD11b | PE | M1/70 | 12-0112 | eBioscience |
| Anti mouse CD170/SiglecF | PerCP-efluor710 | 1RNM44N | 46-1702 | eBioscience |
| Anti mouse Ly6G | APC | RB6-8C5 | 17-5931 | eBioscience |
| Anti human HLA-DR | FITC | AC122 | 130-113-401 | Miltenyi Biotec |
| Anti human CD86 | PE | FM95 | 130-094-877 | Miltenyi Biotec |
| Anti human CD83 | APC | REA714 | 130-094-186 | Miltenyi Biotec |

**Supplementary Table 2 Dilutions of samples and detection antibodies for ELISA**

| Antibody | Reference | Company | Dilution  conjugate | Dilution sample |
| --- | --- | --- | --- | --- |
| *B. tropicalis* specific antibodies | | | | |
| Biotin Anti mouse IgE | 13-5992-82 | eBioscience | 1:1000 | 1:10 |
| Biotin Rat Anti mouse IgG1 | 553441 | BD Pharmigen | 1:10000 | 1:80000 |
| Biotin Rat Anti mouse IgG2a | 550332 | BD Pharmigen | 1:1000 | 1:320 |
| Total antibodies | | | | |
| Mouse IgG1 Ready SET-Go | 88-50410 | eBioscience | Recommended by the manufacturer | 1:20000 |
| Mouse IgG2a Ready SET-Go | 88-50420 | eBioscience | Recommended by the manufacturer | 1:20000 |
| Mouse IgG Total Ready SET-Go | 88-50-400 | eBioscience | Recommended by the manufacturer | 1:100000 |

**Supplementary Figures**

**
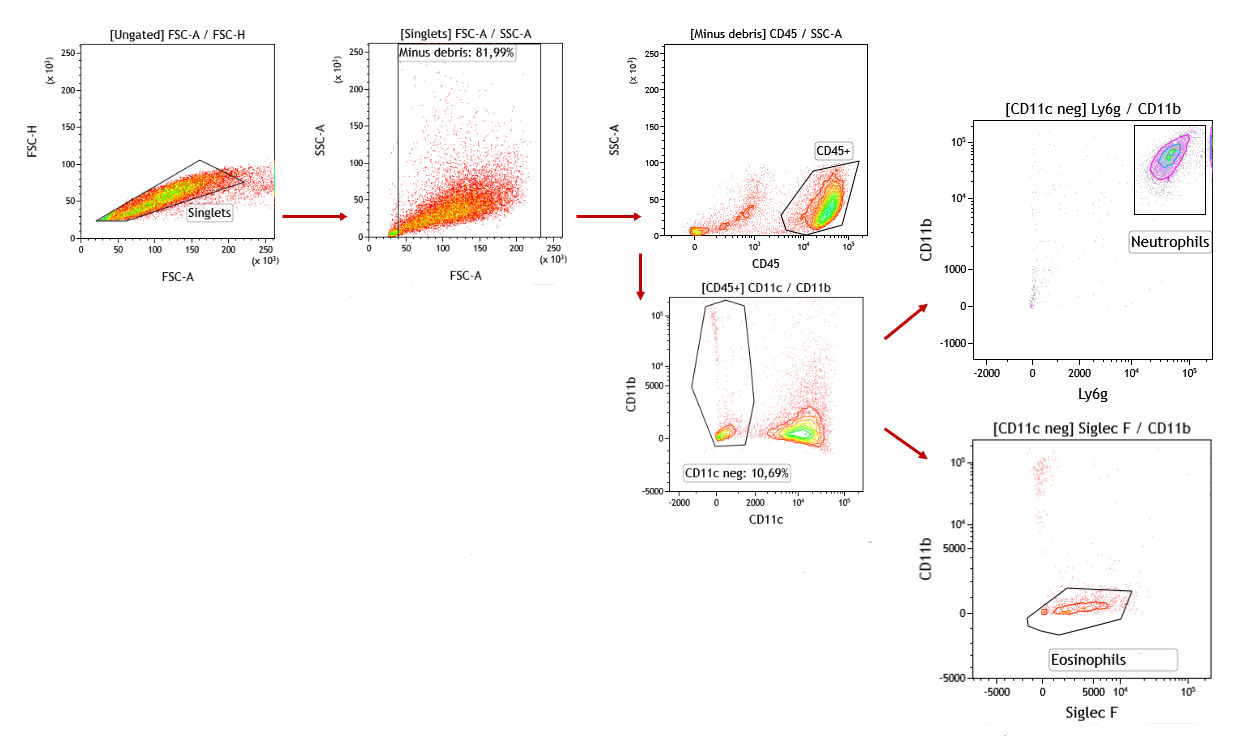
**

**Supplementary Figure 1.** Gating strategy to identify immune cells in BAL**.** After the exclusion of doublets and debris, immune cells were identified using the pan-hematopoietic marker CD45. Neutrophils were identified as Ly6G+ CD11b+ CD11c- and eosinophils as Siglec F+ CD11c- Ly6G-. Cell frequencies were calculated using live, single, CD45+ cells as denominator.


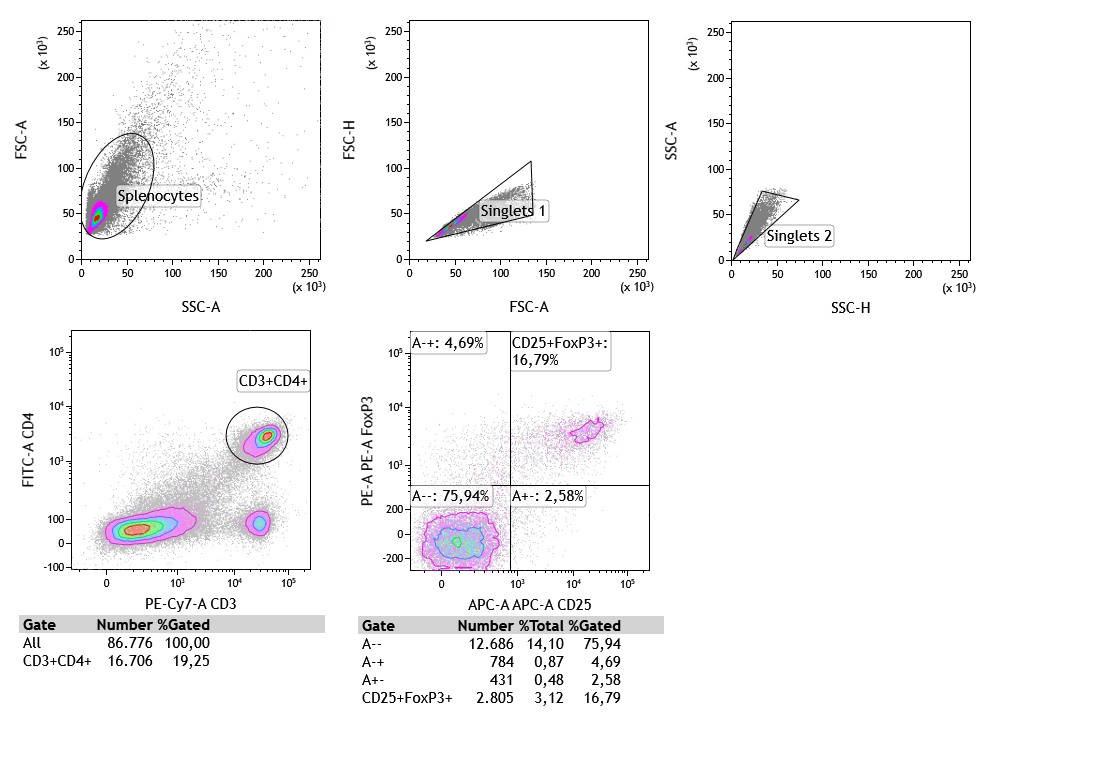


**Supplementary Figure 2**. Gating strategy defining splenic mouse Tregs. Data are representative of 3 independent experiments, with 6-7 mice per group. Forward‐scatter height (FSC‐H) versus forward‐scatter area (FSC‐A) plot for doublet exclusions, followed by a FSC‐A versus side‐scatter area (SSC‐A) plot to delimitate the lymphocyte region. Then, a CD3 versus CD4 plot was used to gate CD4+ T cells, and percentages of CD25+forkhead box protein 3 (FoxP3+).


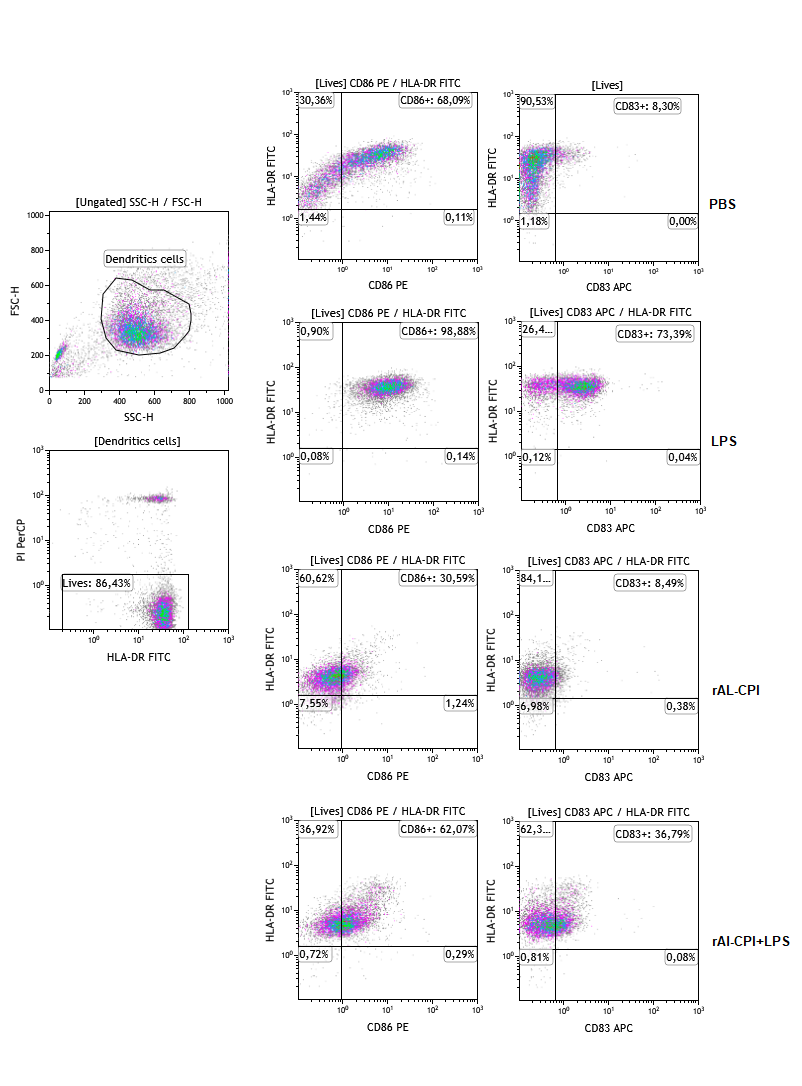


**Supplementary Figure 3.** Generation of functional human Monocyte dendritic cells. Monocytes were cultured in medium containing GM-CSF, IL-4 for seven days, either non-treated or treated LPS were examined for the expression of mature DC markers by a flow cytometry. Dead cells were excluded by using propidium iodide staining. The percentages of the positive cells in the gated population were shown in the dot plots for HLA-DR high expression and CD86 or CD83 maturation markers. Data shown are representative using cells from five different donors. Mean intensity fluorescence values (MFI) of CD83 and CD86 reported in the manuscript were calculated on live+HLA-DR+ cells.


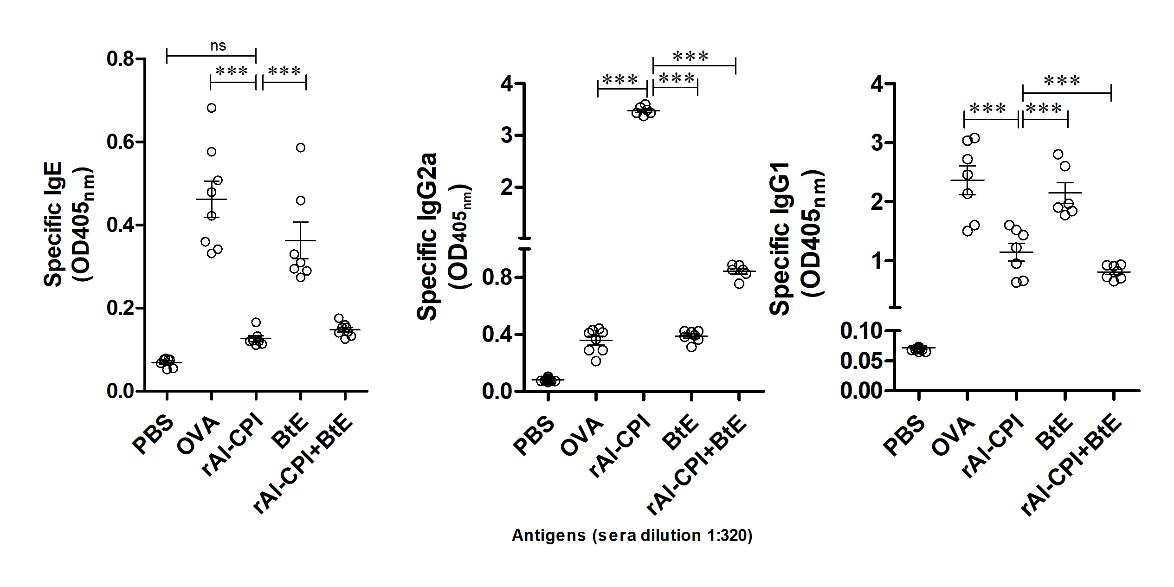


**Supplementary Figure 4.** Antibodies IgG2a and IgG1 produced by rAl-CPI only administration. Data presented are means values ± SEM for 6-8 mice per group; representative data from three independent experiments. (One-way ANOVA + Bonferroni). Significance thresholds are indicated as follows: *p≤0.05; **p≤0.01, ***p≤ 0.001.





**Supplementary Figure 5.** Systemic cytokine responses to IL-10R blocking. Cytokines in spleen cell cultures restimulated in vitro with BtE. The mean values ± SEM are shown for 5-7 mice per group; (Anova one way + Bonferroni). Significance thresholds are indicated as follows: *p≤0.05; **p≤0.01, ***p≤ 0.001.


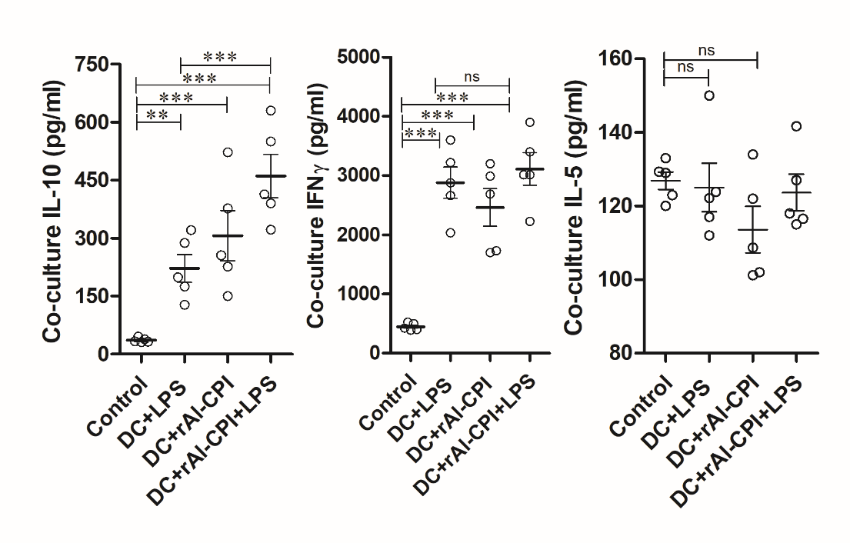


**Supplementary Figure 6.** Cytokines detected in the co-culture of naive T lymphocytes and dendritic cells previously stimulated with LPS, rAl-CPI or rAl-CPI+LPS. Data presented are means of duplicate and from five different donors. Significance thresholds are indicated as follows; p*< 0.05; ***p< 0.01, ***p< 0.001, are indicated.
